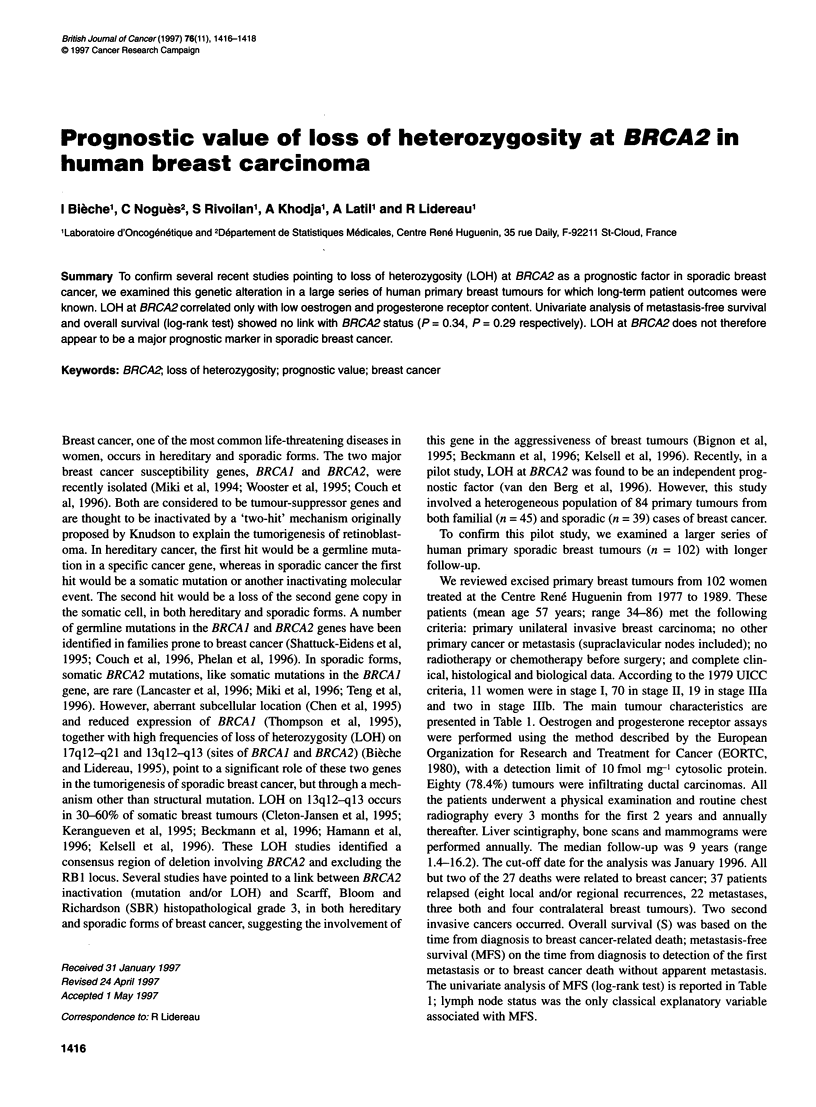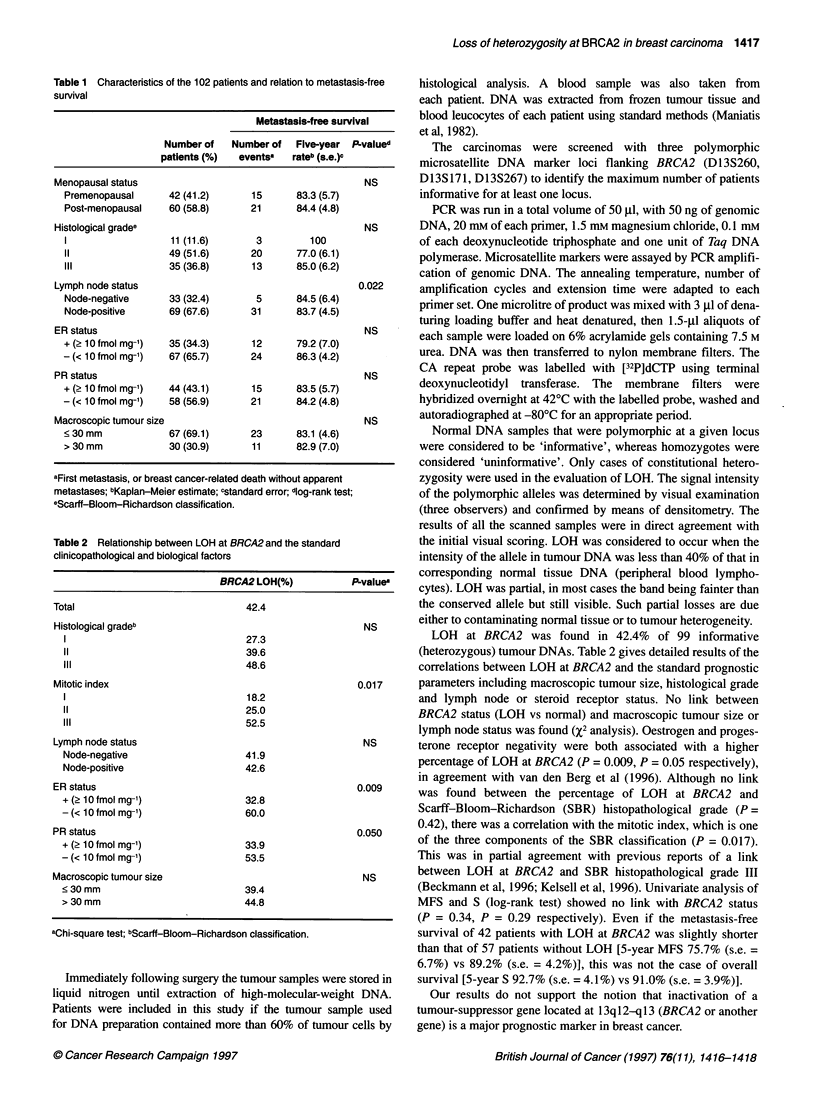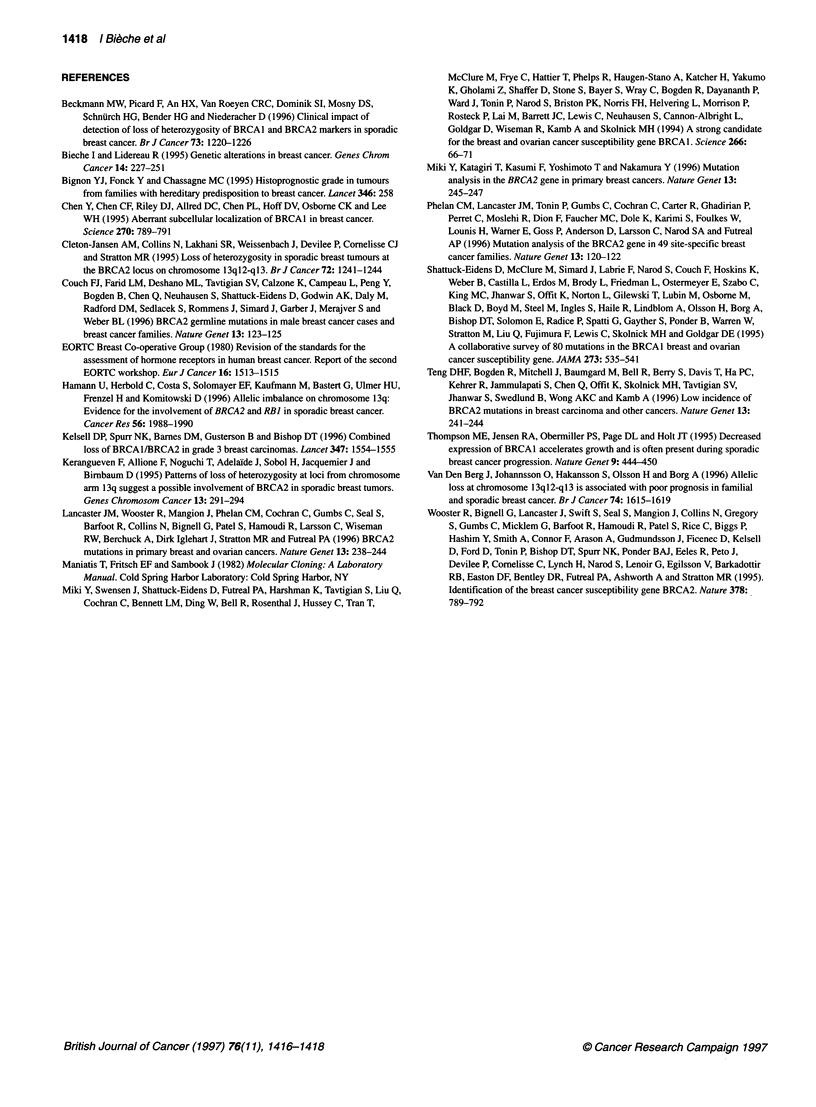# Prognostic value of loss of heterozygosity at BRCA2 in human breast carcinoma.

**DOI:** 10.1038/bjc.1997.572

**Published:** 1997

**Authors:** I. BiÃ¨che, C. NoguÃ¨s, S. Rivoilan, A. Khodja, A. Latil, R. Lidereau

**Affiliations:** Laboratoire d'OncogÃ©nÃ©tique, Centre RenÃ© Huguenin, St-Cloud, France.

## Abstract

To confirm several recent studies pointing to loss of heterozygosity (LOH) at BRCA2 as a prognostic factor in sporadic breast cancer, we examined this genetic alteration in a large series of human primary breast tumours for which long-term patient outcomes were known. LOH at BRCA2 correlated only with low oestrogen and progesterone receptor content. Univariate analysis of metastasis-free survival and overall survival (log-rank test) showed no link with BRCA2 status (P = 0.34, P = 0.29 respectively). LOH at BRCA2 does not therefore appear to be a major prognostic marker in sporadic breast cancer.


British Journal of Cancer (1997) 76(11), 1416–1418
© 1997 Cancer Research Campaign

### Prognostic value of loss of heterozygosity at *BRCA2* in human breast carcinoma

**I Bièche[1], C Nogués[2], S Rivoilan[1], A Khodja[1], A Latil[1] and R Lidereau[1]**

[1]Laboratoire d'Oncogénétique and [2]Département de Statistiques Médicales, Centre René Huguenin, 35 rue Daily, F-92211 St-Cloud, France

**Summary** To confirm several recent studies pointing to loss of heterozygosity (LOH) at *BRCA2* as a prognostic factor in sporadic breast cancer, we examined this genetic alteration in a large series of human primary breast tumours for which long-term patient outcomes were known. LOH at *BRCA2* correlated only with low oestrogen and progesterone receptor content. Univariate analysis of metastasis-free survival and overall survival (log-rank test) showed no link with *BRCA2* status ($P = 0.34$, $P = 0.29$ respectively). LOH at *BRCA2* does not therefore appear to be a major prognostic marker in sporadic breast cancer.

**Keywords:** *BRCA2*; loss of heterozygosity; prognostic value; breast cancer

Breast cancer, one of the most common life-threatening diseases in women, occurs in hereditary and sporadic forms. The two major breast cancer susceptibility genes, *BRCA1* and *BRCA2*, were recently isolated (Miki et al, 1994; Wooster et al, 1995; Couch et al, 1996). Both are considered to be tumour-suppressor genes and are thought to be inactivated by a 'two-hit' mechanism originally proposed by Knudson to explain the tumorigenesis of retinoblastoma. In hereditary cancer, the first hit would be a germline mutation in a specific cancer gene, whereas in sporadic cancer the first hit would be a somatic mutation or another inactivating molecular event. The second hit would be a loss of the second gene copy in the somatic cell, in both hereditary and sporadic forms. A number of germline mutations in the *BRCA1* and *BRCA2* genes have been identified in families prone to breast cancer (Shattuck-Eidens et al, 1995; Couch et al, 1996, Phelan et al, 1996). In sporadic forms, somatic *BRCA2* mutations, like somatic mutations in the *BRCA1* gene, are rare (Lancaster et al, 1996; Miki et al, 1996; Teng et al, 1996). However, aberrant subcellular location (Chen et al, 1995) and reduced expression of *BRCA1* (Thompson et al, 1995), together with high frequencies of loss of heterozygosity (LOH) on 17q12–q21 and 13q12–q13 (sites of *BRCA1* and *BRCA2*) (Bièche and Lidereau, 1995), point to a significant role of these two genes in the tumorigenesis of sporadic breast cancer, but through a mechanism other than structural mutation. LOH on 13q12–q13 occurs in 30–60% of somatic breast tumours (Cleton-Jansen et al, 1995; Kerangueven et al, 1995; Beckmann et al, 1996; Hamann et al, 1996; Kelsell et al, 1996). These LOH studies identified a consensus region of deletion involving *BRCA2* and excluding the RB1 locus. Several studies have pointed to a link between *BRCA2* inactivation (mutation and/or LOH) and Scarff, Bloom and Richardson (SBR) histopathological grade 3, in both hereditary and sporadic forms of breast cancer, suggesting the involvement of this gene in the aggressiveness of breast tumours (Bignon et al, 1995; Beckmann et al, 1996; Kelsell et al, 1996). Recently, in a pilot study, LOH at *BRCA2* was found to be an independent prognostic factor (van den Berg et al, 1996). However, this study involved a heterogeneous population of 84 primary tumours from both familial ($n = 45$) and sporadic ($n = 39$) cases of breast cancer.

To confirm this pilot study, we examined a larger series of human primary sporadic breast tumours ($n = 102$) with longer follow-up.

We reviewed excised primary breast tumours from 102 women treated at the Centre René Huguenin from 1977 to 1989. These patients (mean age 57 years; range 34–86) met the following criteria: primary unilateral invasive breast carcinoma; no other primary cancer or metastasis (supraclavicular nodes included); no radiotherapy or chemotherapy before surgery; and complete clinical, histological and biological data. According to the 1979 UICC criteria, 11 women were in stage I, 70 in stage II, 19 in stage IIIa and two in stage IIIb. The main tumour characteristics are presented in Table 1. Oestrogen and progesterone receptor assays were performed using the method described by the European Organization for Research and Treatment for Cancer (EORTC, 1980), with a detection limit of 10 fmol $mg^{-1}$ cytosolic protein. Eighty (78.4%) tumours were infiltrating ductal carcinomas. All the patients underwent a physical examination and routine chest radiography every 3 months for the first 2 years and annually thereafter. Liver scintigraphy, bone scans and mammograms were performed annually. The median follow-up was 9 years (range 1.4–16.2). The cut-off date for the analysis was January 1996. All but two of the 27 deaths were related to breast cancer; 37 patients relapsed (eight local and/or regional recurrences, 22 metastases, three both and four contralateral breast tumours). Two second invasive cancers occurred. Overall survival (S) was based on the time from diagnosis to breast cancer-related death; metastasis-free survival (MFS) on the time from diagnosis to detection of the first metastasis or to breast cancer death without apparent metastasis. The univariate analysis of MFS (log-rank test) is reported in Table 1; lymph node status was the only classical explanatory variable associated with MFS.

*Received 31 January 1997*
*Revised 24 April 1997*
*Accepted 1 May 1997*

*Correspondence to:* R Lidereau

**Table 1** Characteristics of the 102 patients and relation to metastasis-free survival

| | Number of patients (%) | Metastasis-free survival | | |
|---|---|---|---|---|
| | | Number of events[a] | Five-year rate[b] (s.e.)[c] | *P*-value[d] |
| Menopausal status | | | | NS |
| Premenopausal | 42 (41.2) | 15 | 83.3 (5.7) | |
| Post-menopausal | 60 (58.8) | 21 | 84.4 (4.8) | |
| Histological grade[e] | | | | NS |
| I | 11 (11.6) | 3 | 100 | |
| II | 49 (51.6) | 20 | 77.0 (6.1) | |
| III | 35 (36.8) | 13 | 85.0 (6.2) | |
| Lymph node status | | | | 0.022 |
| Node-negative | 33 (32.4) | 5 | 84.5 (6.4) | |
| Node-positive | 69 (67.6) | 31 | 83.7 (4.5) | |
| ER status | | | | NS |
| + (≥ 10 fmol mg$^{-1}$) | 35 (34.3) | 12 | 79.2 (7.0) | |
| – (< 10 fmol mg$^{-1}$) | 67 (65.7) | 24 | 86.3 (4.2) | |
| PR status | | | | NS |
| + (≥ 10 fmol mg$^{-1}$) | 44 (43.1) | 15 | 83.5 (5.7) | |
| – (< 10 fmol mg$^{-1}$) | 58 (56.9) | 21 | 84.2 (4.8) | |
| Macroscopic tumour size | | | | NS |
| ≤ 30 mm | 67 (69.1) | 23 | 83.1 (4.6) | |
| > 30 mm | 30 (30.9) | 11 | 82.9 (7.0) | |

[a]First metastasis, or breast cancer-related death without apparent metastases; [b]Kaplan–Meier estimate; [c]standard error; [d]log-rank test; [e]Scarff–Bloom–Richardson classification.

**Table 2** Relationship between LOH at *BRCA2* and the standard clinicopathological and biological factors

| | *BRCA2* LOH(%) | *P*-value[a] |
|---|---|---|
| Total | 42.4 | |
| Histological grade[b] | | NS |
| I | 27.3 | |
| II | 39.6 | |
| III | 48.6 | |
| Mitotic index | | 0.017 |
| I | 18.2 | |
| II | 25.0 | |
| III | 52.5 | |
| Lymph node status | | NS |
| Node-negative | 41.9 | |
| Node-positive | 42.6 | |
| ER status | | 0.009 |
| + (≥ 10 fmol mg$^{-1}$) | 32.8 | |
| – (< 10 fmol mg$^{-1}$) | 60.0 | |
| PR status | | 0.050 |
| + (≥ 10 fmol mg$^{-1}$) | 33.9 | |
| – (< 10 fmol mg$^{-1}$) | 53.5 | |
| Macroscopic tumour size | | NS |
| ≤ 30 mm | 39.4 | |
| > 30 mm | 44.8 | |

[a]Chi-square test; [b]Scarff–Bloom–Richardson classification.

Immediately following surgery the tumour samples were stored in liquid nitrogen until extraction of high-molecular-weight DNA. Patients were included in this study if the tumour sample used for DNA preparation contained more than 60% of tumour cells by histological analysis. A blood sample was also taken from each patient. DNA was extracted from frozen tumour tissue and blood leucocytes of each patient using standard methods (Maniatis et al, 1982).

The carcinomas were screened with three polymorphic microsatellite DNA marker loci flanking *BRCA2* (D13S260, D13S171, D13S267) to identify the maximum number of patients informative for at least one locus.

PCR was run in a total volume of 50 µl, with 50 ng of genomic DNA, 20 mM of each primer, 1.5 mM magnesium chloride, 0.1 mM of each deoxynucleotide triphosphate and one unit of *Taq* DNA polymerase. Microsatellite markers were assayed by PCR amplification of genomic DNA. The annealing temperature, number of amplification cycles and extension time were adapted to each primer set. One microlitre of product was mixed with 3 µl of denaturing loading buffer and heat denatured, then 1.5-µl aliquots of each sample were loaded on 6% acrylamide gels containing 7.5 M urea. DNA was then transferred to nylon membrane filters. The CA repeat probe was labelled with [$^{32}$P]dCTP using terminal deoxynucleotidyl transferase. The membrane filters were hybridized overnight at 42°C with the labelled probe, washed and autoradiographed at –80°C for an appropriate period.

Normal DNA samples that were polymorphic at a given locus were considered to be 'informative', whereas homozygotes were considered 'uninformative'. Only cases of constitutional heterozygosity were used in the evaluation of LOH. The signal intensity of the polymorphic alleles was determined by visual examination (three observers) and confirmed by means of densitometry. The results of all the scanned samples were in direct agreement with the initial visual scoring. LOH was considered to occur when the intensity of the allele in tumour DNA was less than 40% of that in corresponding normal tissue DNA (peripheral blood lymphocytes). LOH was partial, in most cases the band being fainter than the conserved allele but still visible. Such partial losses are due either to contaminating normal tissue or to tumour heterogeneity.

LOH at *BRCA2* was found in 42.4% of 99 informative (heterozygous) tumour DNAs. Table 2 gives detailed results of the correlations between LOH at *BRCA2* and the standard prognostic parameters including macroscopic tumour size, histological grade and lymph node or steroid receptor status. No link between *BRCA2* status (LOH vs normal) and macroscopic tumour size or lymph node status was found ($\chi^2$ analysis). Oestrogen and progesterone receptor negativity were both associated with a higher percentage of LOH at *BRCA2* ($P$ = 0.009, $P$ = 0.05 respectively), in agreement with van den Berg et al (1996). Although no link was found between the percentage of LOH at *BRCA2* and Scarff–Bloom–Richardson (SBR) histopathological grade ($P$ = 0.42), there was a correlation with the mitotic index, which is one of the three components of the SBR classification ($P$ = 0.017). This was in partial agreement with previous reports of a link between LOH at *BRCA2* and SBR histopathological grade III (Beckmann et al, 1996; Kelsell et al, 1996). Univariate analysis of MFS and S (log-rank test) showed no link with *BRCA2* status ($P$ = 0.34, $P$ = 0.29 respectively). Even if the metastasis-free survival of 42 patients with LOH at *BRCA2* was slightly shorter than that of 57 patients without LOH [5-year MFS 75.7% (s.e. = 6.7%) vs 89.2% (s.e. = 4.2%)], this was not the case of overall survival [5-year S 92.7% (s.e. = 4.1%) vs 91.0% (s.e. = 3.9%)].

Our results do not support the notion that inactivation of a tumour-suppressor gene located at 13q12–q13 (*BRCA2* or another gene) is a major prognostic marker in breast cancer.

© Cancer Research Campaign 1997 

### REFERENCES

Beckmann MW, Picard F, An HX, Van Roeyen CRC, Dominik SI, Mosny DS, Schnürch HG, Bender HG and Niederacher D (1996) Clinical impact of detection of loss of heterozygosity of BRCA1 and BRCA2 markers in sporadic breast cancer. *Br J Cancer* **73:** 1220–1226

Bieche I and Lidereau R (1995) Genetic alterations in breast cancer. *Genes Chrom Cancer* **14:** 227–251

Bignon YJ, Fonck Y and Chassagne MC (1995) Histoprognostic grade in tumours from families with hereditary predisposition to breast cancer. *Lancet* **346:** 258

Chen Y, Chen CF, Riley DJ, Allred DC, Chen PL, Hoff DV, Osborne CK and Lee WH (1995) Aberrant subcellular localization of BRCA1 in breast cancer. *Science* **270:** 789–791

Cleton-Jansen AM, Collins N, Lakhani SR, Weissenbach J, Devilee P, Cornelisse CJ and Stratton MR (1995) Loss of heterozygosity in sporadic breast tumours at the BRCA2 locus on chromosome 13q12-q13. *Br J Cancer* **72:** 1241–1244

Couch FJ, Farid LM, Deshano ML, Tavtigian SV, Calzone K, Campeau L, Peng Y, Bogden B, Chen Q, Neuhausen S, Shattuck-Eidens D, Godwin AK, Daly M, Radford DM, Sedlacek S, Rommens J, Simard J, Garber J, Merajver S and Weber BL (1996) BRCA2 germline mutations in male breast cancer cases and breast cancer families. *Nature Genet* **13:** 123–125

EORTC Breast Co-operative Group (1980) Revision of the standards for the assessment of hormone receptors in human breast cancer. Report of the second EORTC workshop. *Eur J Cancer* **16:** 1513–1515

Hamann U, Herbold C, Costa S, Solomayer EF, Kaufmann M, Bastert G, Ulmer HU, Frenzel H and Komitowski D (1996) Allelic imbalance on chromosome 13q: Evidence for the involvement of *BRCA2* and *RB1* in sporadic breast cancer. *Cancer Res* **56:** 1988–1990

Kelsell DP, Spurr NK, Barnes DM, Gusterson B and Bishop DT (1996) Combined loss of BRCA1/BRCA2 in grade 3 breast carcinomas. *Lancet* **347:** 1554–1555

Kerangueven F, Allione F, Noguchi T, Adelaïde J, Sobol H, Jacquemier J and Birnbaum D (1995) Patterns of loss of heterozygosity at loci from chromosome arm 13q suggest a possible involvement of BRCA2 in sporadic breast tumors. *Genes Chromosom Cancer* **13:** 291–294

Lancaster JM, Wooster R, Mangion J, Phelan CM, Cochran C, Gumbs C, Seal S, Barfoot R, Collins N, Bignell G, Patel S, Hamoudi R, Larsson C, Wiseman RW, Berchuck A, Dirk Iglehart J, Stratton MR and Futreal PA (1996) BRCA2 mutations in primary breast and ovarian cancers. *Nature Genet* **13:** 238–244

Maniatis T, Fritsch EF and Sambook J (1982) *Molecular Cloning: A Laboratory Manual.* Cold Spring Harbor Laboratory: Cold Spring Harbor, NY

Miki Y, Swensen J, Shattuck-Eidens D, Futreal PA, Harshman K, Tavtigian S, Liu Q, Cochran C, Bennett LM, Ding W, Bell R, Rosenthal J, Hussey C, Tran T, McClure M, Frye C, Hattier T, Phelps R, Haugen-Stano A, Katcher H, Yakumo K, Gholami Z, Shaffer D, Stone S, Bayer S, Wray C, Bogden R, Dayananth P, Ward J, Tonin P, Narod S, Briston PK, Norris FH, Helvering L, Morrison P, Rosteck P, Lai M, Barrett JC, Lewis C, Neuhausen S, Cannon-Albright L, Goldgar D, Wiseman R, Kamb A and Skolnick MH (1994) A strong candidate for the breast and ovarian cancer susceptibility gene BRCA1. *Science* **266:** 66–71

Miki Y, Katagiri T, Kasumi F, Yoshimoto T and Nakamura Y (1996) Mutation analysis in the *BRCA2* gene in primary breast cancers. *Nature Genet* **13:** 245–247

Phelan CM, Lancaster JM, Tonin P, Gumbs C, Cochran C, Carter R, Ghadirian P, Perret C, Moslehi R, Dion F, Faucher MC, Dole K, Karimi S, Foulkes W, Lounis H, Warner E, Goss P, Anderson D, Larsson C, Narod SA and Futreal AP (1996) Mutation analysis of the BRCA2 gene in 49 site-specific breast cancer families. *Nature Genet* **13:** 120–122

Shattuck-Eidens D, McClure M, Simard J, Labrie F, Narod S, Couch F, Hoskins K, Weber B, Castilla L, Erdos M, Brody L, Friedman L, Ostermeyer E, Szabo C, King MC, Jhanwar S, Offit K, Norton L, Gilewski T, Lubin M, Osborne M, Black D, Boyd M, Steel M, Ingles S, Haile R, Lindblom A, Olsson H, Borg A, Bishop DT, Solomon E, Radice P, Spatti G, Gayther S, Ponder B, Warren W, Stratton M, Liu Q, Fujimura F, Lewis C, Skolnick MH and Goldgar DE (1995) A collaborative survey of 80 mutations in the BRCA1 breast and ovarian cancer susceptibility gene. *JAMA* **273:** 535–541

Teng DHF, Bogden R, Mitchell J, Baumgard M, Bell R, Berry S, Davis T, Ha PC, Kehrer R, Jammulapati S, Chen Q, Offit K, Skolnick MH, Tavtigian SV, Jhanwar S, Swedlund B, Wong AKC and Kamb A (1996) Low incidence of BRCA2 mutations in breast carcinoma and other cancers. *Nature Genet* **13:** 241–244

Thompson ME, Jensen RA, Obermiller PS, Page DL and Holt JT (1995) Decreased expression of BRCA1 accelerates growth and is often present during sporadic breast cancer progression. *Nature Genet* **9:** 444–450

Van Den Berg J, Johannsson O, Hakansson S, Olsson H and Borg A (1996) Allelic loss at chromosome 13q12-q13 is associated with poor prognosis in familial and sporadic breast cancer. *Br J Cancer* **74:** 1615–1619

Wooster R, Bignell G, Lancaster J, Swift S, Seal S, Mangion J, Collins N, Gregory S, Gumbs C, Micklem G, Barfoot R, Hamoudi R, Patel S, Rice C, Biggs P, Hashim Y, Smith A, Connor F, Arason A, Gudmundsson J, Ficenec D, Kelsell D, Ford D, Tonin P, Bishop DT, Spurr NK, Ponder BAJ, Eeles R, Peto J, Devilee P, Cornelisse C, Lynch H, Narod S, Lenoir G, Egilsson V, Barkadottir RB, Easton DF, Bentley DR, Futreal PA, Ashworth A and Stratton MR (1995). Identification of the breast cancer susceptibility gene BRCA2. *Nature* **378:** 789–792

*© Cancer Research Campaign 1997*